# High-Dimensional and Multi-Intensity One-Photon-Interference Quantum Secure Direct Communication

**DOI:** 10.3390/e27040332

**Published:** 2025-03-22

**Authors:** Yu-Ting Lei, Xiang-Jie Li, Xing-Bo Pan, Yun-Rong Zhang, Gui-Lu Long

**Affiliations:** 1 State Key Laboratory of Low-Dimensional Quantum Physics and Department of Physics, Tsinghua University, Beijing 100084, China; 2 Future Research Lab, China Mobile Research Institute, Beijing 100053, China; 3 Beijing Academy of Quantum Information Sciences, Beijing 100193, China; 4 Frontier Science Center for Quantum Information, Beijing 100084, China; 5 Beijing National Research Center for Information Science and Technology, Beijing 100084, China

**Keywords:** quantum information, quantum communication, quantum secure direct communication, one-photon-interference quantum communication

## Abstract

As a novel paradigm in quantum communication, quantum secure direct communication (QSDC) enables secure, reliable, and deterministic information transmission, leveraging the principles of quantum mechanics. One-photon-interference QSDC is particularly attractive because it mitigates the vulnerabilities in measurement devices while extending transmission distances. In this paper, we propose a high-dimensional one-photon-interference QSDC protocol that exploits the advantages of high-dimensional encoding in the phase of weak coherent pluses to further enhance transmission distances and improve secrecy channel capacity. The security of this protocol is analyzed using quantum wiretap channel theory, and its resistance to common quantum threats is discussed. Numerical simulations demonstrate that our protocol outperforms its predecessor in terms of its secrecy capacity and extends the maximum communication distance achievable up to 494 km, which is over 13% longer than the two-dimensional case, effectively doubling the transmission length of traditional protocols. These improvements highlight the protocol’s potential for use in quantum communication applications in this era of frequent data breaches and information leaks.

## 1. Introduction

The last two decades have witnessed the rapid development of quantum communication, which has garnered extensive attention due to its high security, guaranteed by the laws of quantum physics. One typical form of quantum communication is quantum key distribution (QKD), which provides secure key agreements between remote parties. Starting with Bennett and Brassard’s pioneering BB84 scheme [1] and the very first entanglement-based protocols, E91 [2] and BBM92 [3], QKD has evolved significantly over the years, and its security has been theoretically proven [4,5,6]. Early efforts aimed to bridge the gap between theoretical security promises and practical implementations, exemplified by the decoy-state method [7,8,9], which mitigates photon-number-splitting (PNS) attacks and enables a high secret key rate even with a practical weak coherent source instead of an ideal single-photon source. To address vulnerabilities arising from detector-side loopholes, a measurement-device-independent QKD (MDI-QKD) [10,11,12] has been proposed to eliminate the security risks associated with measurement-device imperfections in legitimate users. On the other hand, quantum secure direct communication (QSDC) has rapidly become a key paradigm of quantum cryptography. It originates from the seminal work by Long and Liu [13], which demonstrated the possibility of direct secret transmission in quantum channels, while subsequent protocols have extended their framework to incorporate various quantum resources, including polarizations in back-and-forth single photons [14]; orbital angular momentum states [15]; hyperentangled states [16]; high-dimensional optical degrees of freedom in both time and phase [17]; quadrature components, which are commonly used in continuous-variable (CV) protocols [18]; and so on. In facing the threats posed by attacks targeting experimental devices, the advent of MDI [19,20,21] and device-independent (DI) [22,23,24] techniques has further enhanced QSDC’s security by incorporating realistic and imperfect implementations into its theoretical framework. QSDC also has the advantage of compatibility with existing Internet infrastructure [25], and simplifies its deployment by trimming the need for the management of pre-distributed keys. Numerous experimental demonstrations in recent years have proved the feasibility of these QKD [26,27,28,29,30] and QSDC [31,32,33,34,35] protocols, thereby increasing their potential for application in future scenarios requiring high levels of security.

The security of QSDC is based on the quantum wiretap channel theory [36,37], taking advantage of channel parameters such as the yield and error rate in transmission. As long as the secrecy channel capacity is non-zero, then there must exist a classical encoding scheme that ensures the secure and reliable transmission of information over a noisy and eavesdropping channel, according to Wyner’s theory [38,39,40].

To further increase the key generation rate and extend the distance of communication, Lucamarini et al. put forward the twin-field QKD (TF-QKD) [41], which replaces the two-photon Bell state measurements in MDI-QKD with single-photon interferences. This allows the key rate to scale with the square root of the channel transmittance, effectively doubling the secure transmission distance compared to prior protocols, and can break the Pirandola–Laurenza–Ottaviani–Banchi (PLOB) bound [42], which was once considered to be unfeasible without quantum repeaters. Thus, this novel feature has led to many research endeavors [43,44,45,46,47,48]. The essential mechanism behind TF-QKD is to exploit the one-photon interference. Inspired by this, one-photon-interference QSDC (OPI-QSDC) [49] is proposed to enhance the practicality and performance of QSDC protocols, while forgoing the need for either ideal single-photon sources, entangled light sources, or quantum memory. Meanwhile, it also possesses the MDI characteristic that mitigates the vulnerabilities in measurement devices.

However, OPI-QSDC employs only two phases for encoding secret information onto weak coherent pulses, leaving room for additional performance enhancement. High-dimensional quantum states not only increase the transmission rate but also enhance the probability of detecting eavesdropping [17,20,26]. In the meantime, by introducing additional bases into the encoding mode when preparing the quantum states to be transmitted, significant reductions in information leakage can be achieved over long distances [47]. Following these works, a high-dimensional one-photon-interference QSDC (HDOPI-QSDC) protocol is proposed in this paper.

The rest of this paper is organized as follows: Section 2 presents a detailed description of the protocol. In Section 3, we analyze the security of the protocol utilizing Wyner’s wiretap theory, and discuss its resistance to several common quantum threats. Section 4 is dedicated to a numerical simulation of our scheme to evaluate its performance compared with two other typical QSDC protocols. A short summary and outlook is given at the end, in Section 5.

## 2. Our Protocol

**Figure 1:**
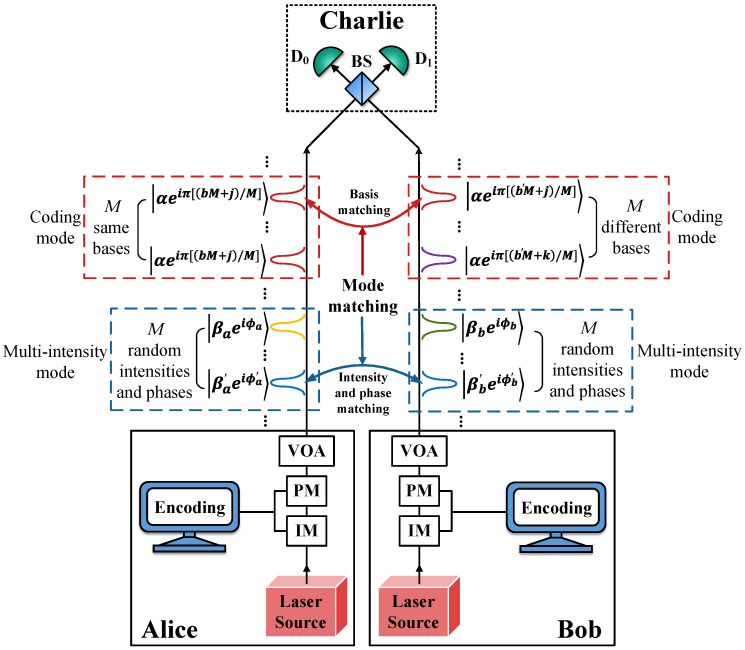
Schematic diagram of the HDOPI-QSDC protocol. BS, 50:50 beam splitter; $D_{0}$ and $D_{1}$, single-photon detectors; VOA, variable optical attenuator; PM, phase modulator; IM, intensity modulator. Red dashed boxes represent the coding modes, and blue dashed boxes represent the multi-intensity modes. In coding modes, Alice’s pulses contain *M* same bases, while Bob’s contain *M* different bases, and the order of these bases is random as well. In multi-intensity modes, Alice and Bob’s *M* pulses contain random intensities and phases, chosen from the sets $\left\{\beta_{0},\beta_{1},\ldots \right\}$ and $\left[0,2\pi \right)$, respectively. j,k∈{0,1,…,M−1} are the bases’ indices and $b,b^{\prime }\in \left\{0,1\right\}$ are the information bits. Charlie conducts one-photon interferences and publishes the untrustworthy measurement results, which are utilized by Alice and Bob to estimate the channel parameters and extract the original secret message.

We assume that Alice and Bob use weak laser pulses with phase locking and have agreed upon a reasonable number of total base slices *M* before completing the following steps. Charlie, an untrusted third party, is in between them, as illustrated in Figure 1. The steps of the HDOPI-QSDC protocol are as follows.

**Step 1: Encoding.** Alice encodes the message to be transmitted into ciphertext using local random numbers shared with Bob. Note that the shared key can be obtained by running the rest of the procedures in this protocol, in which case random numbers are sent instead of the ciphertext. The encoding process includes forward error correction (FEC) coding, secure coding [37], and INCUM (increase capacity using masking) [50]. These processes eliminate the protocol’s reliance on quantum memory, and their details are provided in Appendix A.

**Step 2: State preparation.** Alice and Bob independently select a mode to operate in: coding mode, with a probability of 1−p, or multi-intensity mode, with a probability of *p*, where p≪1. The specifics of the coding mode and multi-intensity mode are detailed below.

*Coding mode:* Alice sends *M* weak coherent states (WCSs) with the same base $|\alpha e^{i\pi (b_{A}/M)}\rangle =|\alpha e^{i\pi [(bM+A)/M]}\rangle$, where b=0 or 1, which is the information bit value and is encoded in the phases as $b_{A}=bM+A\in \left\{0,1,\ldots ,2M-1\right\}$, with A∈{0,1,…,M−1} being the base index and *M* the total number of bases. Bob sends *M* WCSs with different bases $|\alpha e^{i\pi (b_{B}/M)}\rangle \in \left\{|\alpha e^{i\pi (b_{0}/M)}\rangle ,|\alpha e^{i\pi (b_{1}/M)}\rangle ,\ldots ,|\alpha e^{i\pi (b_{M-1}/M)}\rangle \right\}$, where $b_{B}=b^{\prime }M+B\in \left\{0,1,\ldots ,2M-1\right\}$ and $b^{\prime }$ is a random bit in 0 or 1. The order of Bob’s WCSs is random.

*Multi-intensity mode:* Alice and Bob send *M* WCSs with random intensities and random phases $|\beta_{a}e^{i\phi_{a}}\rangle ,|\beta_{b}e^{i\phi_{b}}\rangle$. $\beta_{a}$ and $\beta_{b}$ are randomly selected light intensities in $\left\{\beta_{0},\beta_{1},\ldots \right\}$, and $\phi_{a},\phi_{b}\in \left[0,2\pi \right)$ are random phases.

**Step 3: Charlie’s measurement.** One-photon interferences between pulses from Alice and Bob are conducted by Charlie, and the measurement results are announced on the public channel. Let $D_{0}$ and $D_{1}$ denote the measurement outcomes of the detectors $D_{0}$ and $D_{1}$, respectively; and their values can be set to “0”, indicating a no-click event, or “1”, indicating a click event. Alice and Bob discard the events where no detector clicks or both of them click, retaining only the one-click events, namely $D_{0}⊕D_{1}=1$.

**Step 4: Mode matching.** After all measurements are completed, Alice and Bob publish their selection of modes and retain events where they chose the same mode. In coding mode, Alice and Bob publish the basis information *A* and *B* and retain the events where they choose the same basis. In multi-intensity mode, Alice and Bob publish the intensities $\beta_{a}$ and $\beta_{b}$ and the phase information $\phi_{a}$ and $\phi_{b}$, and then retain the events where $\beta_{a}=\beta_{b}$ and $|\phi_{a}-\phi_{b}|=0$ or π.

**Step 5: Parameter estimation.** Alice and Bob randomly publish some bit values in coding modes to estimate the quantum bit error rate (QBER), and use multi-intensity modes to estimate the amount of information leakage.

**Step 6: Decoding.** Bob decodes the message from the ciphertext. The details of the decoding process are described in Appendix A.

It is important to note that mode mismatches occur with a probability of 2p(1−p), resulting in the possible loss of information transmitted by Alice. This necessitates the use of error-correcting codes during the pre-encoding process.

## 3. Security Analysis

According to quantum wiretap channel theory, when the capacity of the main channel is higher than that of the wiretap channel, a feasible coding scheme can be found that achieves secure and reliable information transmission. We introduce an equivalent entanglement-based protocol, the details of which are given in Appendix B, and analyze its security so as to determine the achievable secrecy capacity *R* of our HDOPI-QSDC protocol. Generally, we know that [51]

(1) $$R=\max\left\{I(A:B)-I(A:E),0\right\},$$

where I(A:B) is the mutual information of Alice and Bob and I(A:E) is the mutual information of Alice and Eve.

Firstly, we consider the achievable secrecy capacity $R^{D_{0}}$ when only detector $D_{0}$ clicks. We assume that Alice and Bob use the *Z* basis to transmit information and the *X* basis to estimate the amount of information leakage. The channels are treated as cascaded channels of a binary erasure channel (BEC) and binary symmetric channel (BSC). The QBER $E_{\mu }^{Z,D_{0}}$ and $E_{\mu }^{X,D_{0}}$, the gain $Q_{\mu }^{D_{0}}$, and the inefficiency function for FEC *f* can be determined through experiments, where $\mu ={\left|\alpha \right|}^{2}$ represents the light intensity of Alice and Bob. Thus, the mutual information I(A:B) satisfies

(2) $$I\left(A:B\right)\leq Q_{\mu }^{D_{0}}\;\cdot \;\left[1-fH_{2}\left(E_{\mu }^{Z,D_{0}}\right)\right],$$

where $H_{2}\left(x\right)$ is the binary entropy function $H_{2}\left(x\right)=-x\log\left(x\right)-\left(1-x\right)\log\left(1-x\right)$. The upper bound of I(A:E) is given by

(3) $$\begin{array}{cc} I(A:E) & \leq Q_{\mu }^{D_{0}}\;\cdot \;H_{2M}\left(E_{\mu }^{X,D_{0}}\right)\\ \; & =-Q_{\mu }^{D_{0}}\sum_{n=0}^{2M-1}\lambda_{0n}\log\left(\lambda_{0n}\right), \end{array}$$

where $\lambda_{0n}$ is defined as

(4) $$\begin{array}{cc} \lambda_{0n}\; & =\frac{1}{Q_{\mu }^{D_{0}}}{\left(\sum_{l=0}^{\infty }C_{2Ml+n}\sqrt{Y_{2Ml+n}^{D_{0}}}\right)}^{2}\\ \; & =\frac{1}{Q_{\mu }^{D_{0}}}{\left(\sum_{l=0}^{\infty }e^{-{\left|\alpha \right|}^{2}}\frac{{\left(\sqrt{2}\alpha \right)}^{2Ml+n}}{\sqrt{(2Ml+n)!}}\sqrt{Y_{2Ml+n}^{D_{0}}}\right)}^{2}, \end{array}$$

and $C_{2Ml+n}$ is the probability amplitude when the number of photons in the channel is 2Ml+n in the event that only detector $D_{0}$ clicks. $Y_{2Ml+n}^{D_{0}}$ represents the yield of the |2Ml+n〉 photon state when only detector $D_{0}$ clicks, and the details are explained in Appendix B. Therefore, we know that

(5) $$R^{D_{0}}=\frac{q}{M}\;\cdot \;Q_{\mu }^{D_{0}}\;\cdot \;\left[1-fH_{2}\left(E_{\mu }^{Z,D_{0}}\right)-H_{2M}\left(E_{\mu }^{X,D_{0}}\right)\right],$$

where q=1−2p(1−p) is the successful rate of mode matching.

The result is similar for the achievable secrecy capacity $R^{D_{1}}$ when only detector $D_{1}$ responds; that is,

(6) $$R^{D_{1}}=\frac{q}{M}\;\cdot \;Q_{\mu }^{D_{1}}\;\cdot \;\left[1-fH_{2}\left(E_{\mu }^{Z,D_{1}}\right)-H_{2M}\left(E_{\mu }^{X,D_{1}}\right)\right].$$

Finally, the total achievable secrecy capacity *R* of the HDOPI-QSDC protocol is given by

(7) $$R=\max\left\{R^{D_{0}},0\right\}+\max\left\{R^{D_{1}},0\right\}.$$

Although the above information-theoretic framework guarantees the feasibility of secure information transmission within our protocol, its resistance to certain well-known attacks should be discussed further. One such attack is the intercept-resend attack, where an adversary Eve attempts to extract information by intercepting, measuring, and then resending quantum states to the intended recipient. However, our protocol is inherently resistant to this attack for the following reasons: First, single-photon interference eliminates the vulnerabilities in direct transmission. Unlike traditional QSDC, Alice and Bob do not exchange qubits directly. Instead, they send phase-encoded weak coherent pulses to a central untrusted relay Charlie, where information is distilled from phase correlations through single-photon interference. If Eve intercepts the photons, she inevitably collapses their quantum states, disrupting the interference and introducing detectable errors in the QBER. Furthermore, intercepting a single path is ineffective since complete information is only reconstructed through interference at the relay. Second, our frame-by-frame encoding scheme (detailed in Appendix A) prevents meaningful data extraction, as each frame carries not only its own ciphertext but also secure keys for subsequent frames. Even in the worst case scenario, where Eve controls both channels and conducts the interference by herself, our encoding strategy not only ensures that this behavior will be immediately perceived by Alice and Bob but also prevents her from obtaining the original secret information, leaving her with only pieces of codewords. Moreover, since this pre-encoding occurs before the secret information is modulated onto quantum states, the scheme effectively leverages the one-time-pad property, significantly reducing the risk of information leakage. Third, INCUM technology further strengthens the protocol’s security by adding another protective layer of masking with locally generated random numbers. This technique restricts Eve’s effective reception rate to Bob’s level, making it even more difficult for her to reconstruct the original information. Together, these mechanisms grant our protocol resistance to intercept-resend attacks.

Another related threat is the PNS attack, where Eve exploits the multi-photon pulses in a practical weak coherent source by splitting off a photon while allowing the remaining photons to reach the legitimate recipient, gaining information without being detected. Our protocol resists this attack through a multi-intensity mode, which functions similarly to the decoy-state method [7,8,9]. Since Eve cannot tell whether a pulse is in encoding mode or multi-intensity mode before her measurement, she cannot selectively attack multi-photon pulses without introducing detectable anomalies. By comparing channel parameters of different light intensities that have different mean photon numbers, Alice and Bob can identify the inconsistencies caused by eavesdropping attempts. As a result, the additional pulses with randomized light intensities and phases protect the multi-photon components of WCS-based encoding schemes, noticeably enhancing their secrecy capacity and ensuring security in the face of PNS attacks.

While quantum communication protocols are theoretically secure under ideal conditions, the measurement devices used in practical systems retain certain loopholes. Imperfections in detectors can be utilized to bypass security guarantees, such as the bright illumination attack [52] and the dead time attack [53]. Our protocol inherits the MDI nature of MDI-QSDC by shifting measurements to an untrusted third party Charlie and relying solely on his results. Because Alice and Bob do not directly receive photons or perform measurements, any inherent imperfections in their detectors do not expose them to vulnerabilities such as side-channel attacks. The security of this protocol relies on quantum interference rather than Charlie’s honesty. Even if his detectors are fully controlled by Eve and are maliciously manipulated, Eve has no chance learning any useful information as it is only derived from post-processed correlations between Alice and Bob, as long as they strictly follow the correct procedures. In addition, as mentioned earlier, parameters such as error rates are carefully monitored, and any unexpected deviations are identified instantly. Although our protocol is not fully DI and may suffer from certain side-channel attacks, such as Trojan-horse attacks on light sources [54], its architecture, which includes placing the measurement in an untrusted location, decouples the security from the trustworthiness of the detectors, making it immune to many common attacks targeting measurement devices.

A final security concern is the assumption of an infinite block length in our analysis. In practical communication systems, Alice and Bob can only send finite numbers of WCS pluses rather than an idealized infinite number. This finite block length introduces statistical fluctuations in the estimation of channel parameters, which further tightens the upper bound of the secrecy channel capacity. Notably, unlike investigations in QKD systems [55,56,57], where random keys are negotiated, QSDC involves the direct transmission of deterministic information, and its performance under these conditions requires specific handling and analysis. While a comprehensive study of finite block length effects and their impact on practical QSDC systems is beyond the scope of this paper, valuable insights on this topic can be found in Refs. [58,59]. In the following discussion, we adhere to the asymptotic limit, assuming an infinite block length.

## 4. Performance

In this section, we analyze the performance of the HDOPI-QSDC protocol. We denote the channel transmittance from Alice and Bob to Charlie as $\eta =\eta_{d}\sqrt{\eta_{c}}$, where $\eta_{d}$ is the detection efficiency and $\eta_{c}$ is the channel loss function. The gain is expressed as

(8) $$Q_{\mu }^{D_{0}}=Q_{\mu }^{D_{1}}=1-e^{-2\eta \mu }+2p_{d}e^{-2\eta \mu },$$

where $p_{d}$ is the rate of dark counts. The QBER is given by

(9) $$E_{\mu }^{X,D_{0}}=E_{\mu }^{X,D_{1}}=\frac{e^{-2\eta \mu }}{Q_{\mu }^{D_{0}}}\left(p_{d}+2\eta \mu \delta \right),$$

where δ is the misalignment error. We assume that Alice and Bob use light pulses with infinite numbers of intensities in the multi-intensity mode, thus the yield of the |2Ml+n〉 photon state is

(10) $$Y_{2Ml+n}^{D_{0}}=Y_{2Ml+n}^{D_{1}}=1-\left(1-2p_{d}\right){\left(1-\eta \right)}^{2Ml+n}.$$

**Figure 2:**
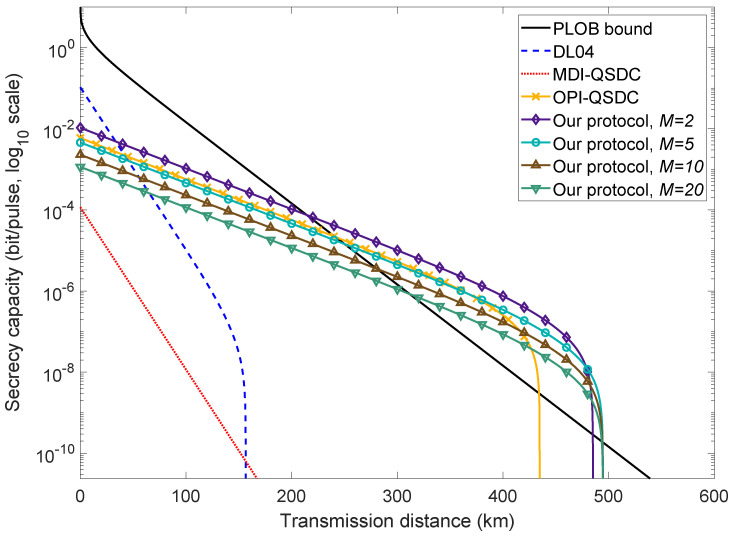
Achievable secrecy channel capacity *R* in $\log_{10}$ scale in terms of transmission distance. The black solid line is the PLOB bound, the red dotted line represents the MDI-QSDC protocol [19] (with INCUM), and the blue dashed line represents the DL04 protocol [14] (with INCUM). The orange line with “x” markers displays the performance of the OPI-QSDC protocol [49], and the purple, cyan, brown, and green lines with hollow markers are our HDOPI-QSDC protocol with M=2, M=5, M=10, and M=20 bases, respectively. The secrecy capacity of our protocol breaks the PLOB bound at 214.17 km (when M=2), and its maximum distance is about 494.58 km (when M=20).

The simulation parameters are as described in Table 1. Following Equation (7), we illustrate the performance of the HDOPI-QSDC protocol and compare it to the PLOB bound [42], the performance of OPI-QSDC (with an optimized intensity u=0.046 as stated in [49]), DL04 ([14], INCUM-enhanced and with ideal sources), and MDI-QSDC ([19], INCUM-enhanced and with ideal sources) in Figure 2. OPI-QSDC can be regarded as an HDOPI-QSDC protocol with M=1, since it uses 0 and π phases in its coding mode. In the case where M=2, our high-dimensional protocol has a higher secrecy capacity and a roughly 50 km longer transmission distance than the original. As *M* increases, its secrecy capacity starts to reduce but its maximum transmission distance grows slightly. When M≥5, its secrecy capacity lags behind that of the original, though within a relatively short range, while its maximum distance outperforms the original by nearly 60 km. The benefit of further increasing *M* diminishes, since the maximum transmission distance hardly lengthens any further and the secrecy channel capacity continues to drop. The determination of a proper *M* should be guided by the specific needs of the system in practical applications. That is to say, M=1, i.e., the original OPI-QSDC, provides a balance between practicality, secrecy capacity, and transmission distance, while a more complicated experimental setup leads to a considerable extension in communication distance and secrecy channel capacity when M=2, 3, or 4.

Table 2 provides a concise comparison between our work and previous studies. It highlights key differences in terms of quantum resources, encoding methodologies, security guarantees, reliance on quantum memory, and performance metrics, thereby clarifying the advantages of our approach.

## 5. Conclusions

In this work, we present a high-dimensional one-photon-interference quantum secure direct communication protocol (HDOPI-QSDC), that generalizes the original one-photon-interference quantum secure direct communication framework to high-dimensional encoding. This advancement results in an enhanced secrecy channel capacity and an extended transmission distance, while maintaining a measurement-device-independent characteristic even though it involves the imperfect measurement devices of legitimate users. The security of the protocol is analyzed utilizing the quantum wiretap channel theory, and the secrecy channel capacity is derived. Furthermore, its resistance to common quantum threats is examined. Numerical simulations demonstrate that the HDOPI-QSDC protocol not only achieves a higher secrecy capacity but also improves the transmission distance by up to approximately 60 km compared to its predecessors, reaching a maximum range of 494 km, which effectively doubles the communication length of traditional protocols. These promising results suggest that our protocol holds potential for future applications, such as intercity quantum communications in government, finance, and healthcare sectors, where its extended range and high capacity could reduce reliance on quantum repeaters. Leveraging the merit of its deterministic information transmission, QSDC integrated with classic or post-quantum cryptography could boost the bandwidth of secure communication and provide an extra layer of protection by transferring only the secret keys via quantum channels, ensuring the hybrid system remains resistant to both quantum and classical adversaries.

However, the proposed protocol is subject to several key constraints that require further investigation. First, our security analysis assumes an infinite block length, which simplifies the derivation of the secrecy capacity by neglecting the statistical fluctuations introduced by finite block lengths in the estimation of channel parameters. In practice, the finite size of the information block leads to tighter bounds and potentially a lower performance. Second, the protocol also presumes an unlimited number of light intensities in the multi-intensity mode. Although three or four intensities should suffice in real-world systems, a detailed capacity analysis of these limited intensity values is needed. Third, the experimental implementation of our protocol faces significant difficulties, due to its heavy reliance on high-precision phase operations. Achieving adequate one-photon interference visibility requires maintaining phase coherence over long distances, between the independent lasers at Alice’s and Bob’s stations. Consequently, the precise synchronization of remote lasers is critical, and active, continuous phase compensation and stabilization is essential to counter environmental disturbances. Moreover, developing high-efficiency and low-noise single-photon detectors remains a substantial challenge. While recent advancements in optical systems [60,61], detector performance [62,63], and protocol optimization [64,65] have shed light on these experimental hurdles with proof-of-principle demonstrations [66,67,68], further innovations will be necessary for the practical deployment of this protocol.

## Figures and Tables

**Table 1:** Key parameter settings of the simulation.

Parameter	Value	Description
ζ	0.2 dB/km	attenuation coefficient
$\eta_{d}$	14.5%	detector efficiency
$p_{d}$	8 × 10^−8^	dark count rate
δ	1.5%	misalignment error
*f*	1.2	FEC efficiency
*u*	0.15	light intensity

**Table 2:** Comparison with other typical QSDC protocols.

	DL04 [14]	MDI-QSDC [19]	OPI-QSDC [49]	Our Protocols
Quantum resources	single photons (ideal) WCSs (practical)	single photons and entanglement pairs	single photons (ideal) WCSs (practical)	single photons (ideal) WCSs (practical)
Encode messages in	polarizations	Bell states	0/π phases	multislice phases
Resistance to measurement-device attacks?	No	Yes	Yes	Yes
Resistance to PNS attacks?	No	No	Yes	Yes
Quantum memory free?	No	No	Yes	Yes
Break PLOB bound?	No	No	Yes	Yes
Approx. secrecy capacity at 100 km (bit/pulse)	1.03 × 10^−5^	1.16 × 10^−8^	5.72 × 10^−4^	1.05 × 10^−3^ (M=2) 1.15 × 10^−4^ (M=20)
Approx. distance at 1 × 10^−10^ bit/pulse secrecy capacity	156.48 km	151.61 km	434.76 km	485.07 km (M=2) 493.94 km (M=20)

**Figure A1:**
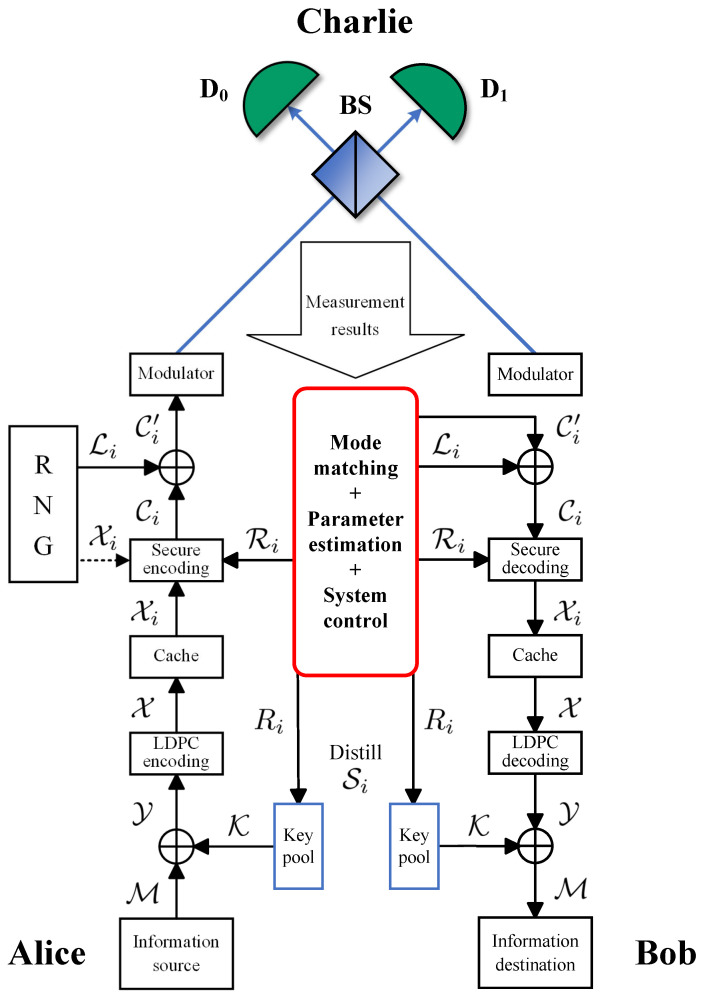
Encoding and decoding processes of the HDOPI-QSDC protocol. BS, 50:50 beam splitter; $D_{0}$ and $D_{1}$, single-photon detectors; RNG, random number generator. The blue arrows indicate the transmission direction of qubits, and the black arrows classical bits. The black dotted arrow is only used in situations where there is an empty cache or key pool and at the beginning of a new round of communication.

## Data Availability

Data are contained within the article.

## Appendix A. Details of Encoding and Decoding Processes

In coding modes, the messages to be transmitted are encoded into frames, and each frame contains the information bits and the raw keys that will be distilled later and used to encode future frames. This removes the need for quantum memory by immediately preparing and sending quantum states without storing them, and it is possible within QSDC because it can examine whether there is eavesdropping in the transmission. Here, we denote $M\in {\left\{0,1\right\}}^{m}$ as plaintext to be transferred from Alice to Bob, $K\in {\left\{0,1\right\}}^{m}$ as keys extracted from a key pool to pre-encrypt the message M, and Y=M⊕K as the ciphertext. Then, a LDPC encoder of length *k* and rate $R_{p}$, satisfying $kR_{p}=m$, is applied for pre-coding, and it outputs $X\in {\left\{0,1\right\}}^{k}$ to a cache. From the cache a sequence $X_{i}\in {\left\{0,1\right\}}^{k_{i}}$ is retrieved, and it forms the input of the secure coding module in the *i*-th frame. However, in the special case that the cache becomes exhausted, $X_{i}$ comes from a random number generator (RNG). Let $R_{i}$ be the secure coding rate of the *i*-th frame and $C_{i}\in {\left\{0,1\right\}}^{n_{C_{i}}}$ be the codeword of $X_{i}$, with length $n_{C_{i}}$. An xor operation is performed on $C_{i}$ with a random bit sequence $L_{i}\in {\left\{0,1\right\}}^{n_{C_{i}}}$. This implements the INCUM method [50], namely by masking the ciphertext $C_{i}$ using local random numbers $L_{i}$, producing $C_{i}^{\prime }=C_{i}⊕L_{i}$, which is then modulated onto quantum states. The encoded quantum state pluses are transmitted to Charlie for measurement. The main channel capacity of the *i*-th frame is denoted as $C_{m_{i}}\equiv \max I_{i}\left(A:B\right)$, and the wiretap channel capacity is $C_{w_{i}}\equiv \max I_{i}\left(A:E\right)$. The achievable secrecy channel capacity $R_{i}\equiv C_{m_{i}}-C_{w_{i}}$ can then be calculated. These parameters must satisfy [37]

(A1) $$\frac{k_{i}}{n_{C_{i}}}\leq R_{i}-C_{w_{i-1}},\;R_{i}<C_{m_{i-1}},$$

to ensure the security of the communication. A diagram of the detailed encoding and decoding process is illustrated in Figure A1, and its steps are as written below.

To initialize a new round of communication, in which case i=1, $X_{1}$ should be picked from an RNG. $k_{1}$ and $R_{1}$ should be properly selected to meet the criteria in Equation (A1). A usable shared key $S_{1}$ with length $n_{C_{1}}\;\cdot \;R_{1}$ can be distilled if $C_{m_{1}}$ and $C_{w_{1}}$ both fulfill Equation (A1) as well.

When i>1 and K is sufficient to use, Alice’s encoding processes and Bob’s decoding processes include the following steps:

(1) Alice uses $K\in {\left\{0,1\right\}}^{m}$ to pre-encrypt $M\in {\left\{0,1\right\}}^{m}$ into the ciphertext Y=M⊕K.
(2) Alice pre-encodes Y into X, which is stored in a cache.
(3) Alice fetches the $k_{i}$-bit length of $X_{i}\in {\left\{0,1\right\}}^{k_{i}}$ from the cache to accomplish secure coding, where the parameters should satisfy Equation (A1) and the output is $C_{i}\in {\left\{0,1\right\}}^{n_{C_{i}}}$.
(4) Alice applies INCUM using a locally generated random bit string $L_{i}$, and obtains $C_{i}^{\prime }=C_{i}⊕L_{i}$.
(5) Alice modulates $C_{i}^{\prime }$ into qubits if she selects the coding mode in **Step 2** of our protocol, otherwise she prepares the multi-intensity mode.
(6) Charlie conducts **Step 3**.
(7) Steps (5) to (6) are repeated until $C_{i}^{\prime }$ is entirely transmitted.
(8) Alice and Bob conduct **Step 4** and **Step 5** and use these parameters to calculate $C_{m_{i}}$, $C_{w_{i}}$, and $R_{i}$. If Equation (A1) is satisfied, a shared key $S_{i}$ could be distilled for future frames.
(9) Steps (3) to (8) are repeated until X is entirely transmitted.
(10) Alice announces random bit values of L in positions where Bob has received information. Bob first applies de-INCUM to obtain $C_{i}=C_{i}^{\prime }⊕L_{i}$ and then decodes $C_{i}$ to $X_{i}$ with a secure coding decoder. After that he obtains Y from a $\left(k,kR_{p}\right)$-LDPC decoder and finally retrieves the original message M=Y⊕K utilizing the shared key K.

## Appendix B. Details of Security Analysis

First, we define two entangled pairs

(A2) $$\begin{array}{c} {|\Phi \left(\alpha \right)\rangle }_{Aa}=\frac{1}{\sqrt{2M}}\sum_{b_{A}=0}^{2M-1}{|b_{A}\rangle }_{A}{|\alpha e^{\pi i\frac{b_{A}}{M}}\rangle }_{a}, \end{array}$$

(A3) $$\begin{array}{c} {|\Phi \left(\alpha \right)\rangle }_{Bb}=\frac{1}{\sqrt{2M}}\sum_{b_{B}=0}^{2M-1}{|b_{B}\rangle }_{B}{|\alpha e^{\pi i\frac{b_{B}}{M}}\rangle }_{b}, \end{array}$$

where the subscripts *A* and *B* of the quantum states indicate that they are retained by Alice and Bob, and *a* and *b* indicate that they are sent to Charlie by Alice and Bob, respectively. The *A* and *B* states are in a codeword space of dimension 2M, thus $b_{A}$ and $b_{B}$ can take values from {0,1,…,2M−1}. Then, we introduce an entanglement-based protocol, taking advantage of these two entangled pairs, which is equivalent in security to our HDOPI-QSDC protocol. For convenience, we refer to this protocol as protocol II and the HDOPI-QSDC protocol as protocol I. Protocol II contains the following steps:

**Step 1: Encoding.** Same as protocol I.

**Step 2’: State preparation.** Alice and Bob select a coding mode with the probability of 1−p or select a multi-intensity mode with *p*, where p≪1.

*Coding mode:* Alice prepares *M* entangled pairs all in the state ${|\Phi \left(\alpha \right)\rangle }_{Aa}$. She sends the photon *a* to Charlie and retains the photon *A* locally. Similarly, Bob prepares *M* entangled pairs ${|\Phi \left(\alpha \right)\rangle }_{Bb}$, sends *b* to Charlie and retains *B*.

*Multi-intensity mode:* Same as protocol I.

**Step 3: Charlie’s measurement.** Same as protocol I.

**Step 3’: Alice and Bob’s measurement.** After Charlie’s measurement, if Alice sent the coding mode photon, she measures the local states *A* in the computational bases. To ensure the equivalence of protocols II and I, we assume that Alice can perform deterministic measurements based on the encoding results. This means that if she wants to send a logical bit 0, and the base index she chooses is also 0, the measurement results of *M* local states will be all ${|0\rangle }_{A}$. If Bob sent the coding mode photon, he measures the local states *B* using *M* different bases.

Note that this step is commutative to **Step 3**. If we exchange these two steps, then protocol II will reduce to protocol I. Thus, the two protocols are equivalent in security.

**Step 4: Mode matching.** Same as protocol I.

**Step 5: Parameter estimation.** Same as protocol I.

**Step 6: Decoding.** Same as protocol I.

Next, we derive the secrecy channel capacity of protocol II. In **Step 2’**, we defined the joint state ${|\phi \rangle }_{AaBb}$ of Alice and Bob, which is

(A4) $$\begin{array}{cc} {|\phi \rangle }_{AaBb} & ={|\Phi \left(\alpha \right)\rangle }_{Aa}⊗{|\Phi \left(\alpha \right)\rangle }_{Bb}\\ & =\frac{1}{\sqrt{2M}}\sum_{b_{A}=0}^{2M-1}{|b_{A}\rangle }_{A}{|\alpha e^{\pi i\frac{b_{A}}{M}}\rangle }_{a}⊗\frac{1}{\sqrt{2M}}\sum_{b_{B}=0}^{2M-1}{|b_{B}\rangle }_{B}{|\alpha e^{\pi i\frac{b_{B}}{M}}\rangle }_{b}\\ & =\frac{1}{2M}\sum_{b_{A},b_{B}}{|b_{A}\rangle }_{A}{|b_{B}\rangle }_{B}{|\alpha e^{\pi i\frac{b_{A}}{M}}\rangle }_{a}{|\alpha e^{\pi i\frac{b_{B}}{M}}\rangle }_{b}. \end{array}$$

Then, Alice and Bob send their photons *a* and *b* to Charlie, respectively.

In **Step 3**, Charlie uses a 50:50 beam splitter to perform single-photon interferences, which result in

(A5) $$\begin{array}{cc} {|\phi \rangle }_{AaBb} & \to {|\phi \rangle }_{ABD_{0}D_{1}}\\ & =\frac{1}{2M}\sum_{b_{A},b_{B}}{|b_{A}\rangle }_{A}{|b_{B}\rangle }_{B}{|\frac{\alpha }{\sqrt{2}}\left(e^{\pi i\frac{b_{A}}{M}}+e^{\pi i\frac{b_{B}}{M}}\right)\rangle }_{D_{0}}{|\frac{\alpha }{\sqrt{2}}\left(e^{\pi i\frac{b_{A}}{M}}-e^{\pi i\frac{b_{B}}{M}}\right)\rangle }_{D_{1}}. \end{array}$$

We first consider the case where only detector $D_{0}$ clicks, i.e., ${|\left(\alpha /\sqrt{2}\right)\left(e^{\pi i(b_{A}/M)}-e^{\pi i(b_{B}/M)}\right)\rangle }_{D_{1}}={|0\rangle }_{D_{1}}$, which implies $b_{B}=b_{A}$. Thus, we obtain

(A6) $$\begin{array}{cc} {|\phi \rangle }_{ABD_{0}D_{1}}= & \frac{1}{2M}\sum_{b_{A}}{|b_{A}\rangle }_{A}{|b_{A}\rangle }_{B}{|\sqrt{2}\alpha e^{\pi i\frac{b_{A}}{M}}\rangle }_{D_{0}}{|0\rangle }_{D_{1}}\\ = & \frac{1}{2M}\sum_{b_{A}}{|b_{A}\rangle }_{A}{|b_{A}\rangle }_{B}\left[e^{-{\left|\alpha \right|}^{2}}\sum_{l=0}^{\infty }\frac{{\left(\sqrt{2}\alpha e^{\pi i\frac{b_{A}}{M}}\right)}^{l}}{\sqrt{l!}}{|l\rangle }_{D_{0}}\right]{|0\rangle }_{D_{1}}\\ = & \frac{e^{-{\left|\alpha \right|}^{2}}}{2M}\left\{\sum_{b_{A}}e^{\pi i\frac{b_{A}\;\cdot \;0}{M}}{|b_{A}\rangle }_{A}{|b_{A}\rangle }_{B}\left[\sum_{l=0}^{\infty }\frac{{\left(\sqrt{2}\alpha \right)}^{2Ml}}{\sqrt{(2Ml)!}}{|2Ml\rangle }_{D_{0}}\right]{|0\rangle }_{D_{1}}\right.\\ & +\sum_{b_{A}}e^{\pi i\frac{b_{A}\;\cdot \;1}{M}}{|b_{A}\rangle }_{A}{|b_{A}\rangle }_{B}\left[\sum_{l=0}^{\infty }\frac{{\left(\sqrt{2}\alpha \right)}^{2Ml+1}}{\sqrt{(2Ml+1)!}}{|2Ml+1\rangle }_{D_{0}}\right]{|0\rangle }_{D_{1}}+\cdots \\ & +\left.\sum_{b_{A}}e^{\pi i\frac{b_{A}\;\cdot \;\left(2M-1\right)}{M}}{|b_{A}\rangle }_{A}{|b_{A}\rangle }_{B}\left[\sum_{l=0}^{\infty }\frac{{\left(\sqrt{2}\alpha \right)}^{2Ml+2M-1}}{\sqrt{(2Ml+2M-1)!}}{|2Ml+2M-1\rangle }_{D_{0}}\right]{|0\rangle }_{D_{1}}\right\}\\ = & \frac{e^{-{\left|\alpha \right|}^{2}}}{\sqrt{2M}}\left[{|\Phi_{00}\rangle }_{AB}\left(\sum_{l=0}^{\infty }\frac{{\left(\sqrt{2}\alpha \right)}^{2Ml}}{\sqrt{(2Ml)!}}{|2Ml\rangle }_{D_{0}}\right){|0\rangle }_{D_{1}}\right.\\ & +{|\Phi_{01}\rangle }_{AB}\left(\sum_{l=0}^{\infty }\frac{{\left(\sqrt{2}\alpha \right)}^{2Ml+1}}{\sqrt{(2Ml+1)!}}{|2Ml+1\rangle }_{D_{0}}\right){|0\rangle }_{D_{1}}+\cdots \\ & +\left.{|\Phi_{0(2M-1)}\rangle }_{AB}\left(\sum_{l=0}^{\infty }\frac{{\left(\sqrt{2}\alpha \right)}^{2Ml+2M-1}}{\sqrt{(2Ml+2M-1)!}}{|2Ml+2M-1\rangle }_{D_{0}}\right){|0\rangle }_{D_{1}}\right], \end{array}$$

where $|\Phi_{mn}\rangle$ is the 2M-dimensional Bell state

(A7) $$|\Phi_{mn}\rangle =\frac{1}{\sqrt{2M}}\sum_{j=0}^{2M-1}e^{\pi i\frac{jn}{M}}|j\rangle |j⊕m\;\left(\mathrm{mod}\;2M\right)\rangle .$$

After Charlie’s measurement, we can trace out systems $D_{0}$ and $D_{1}$ from ${|\phi \rangle }_{ABD_{0}D_{1}}$, and then obtain the joint state of Alice and Bob, which is

(A8) $$\begin{array}{cc} \rho_{AB} & =\sum_{n=0}^{2M-1}\rho_{AB}^{n}\\ & =\frac{1}{Q}\sum_{n=0}^{2M-1}{\left(\sum_{l=0}^{\infty }e^{-{\left|\alpha \right|}^{2}}\frac{{\left(\sqrt{2}\alpha \right)}^{2Ml+n}}{\sqrt{(2Ml+n)!}}\right)}^{2}|\Phi_{0n}\rangle \langle \Phi_{0n}|\\ & \equiv \frac{1}{Q}\sum_{n=0}^{2M-1}{\left(\sum_{l=0}^{\infty }C_{2Ml+n}\right)}^{2}|\Phi_{0n}\rangle \langle \Phi_{0n}|, \end{array}$$

where *Q* is a normalization coefficient.

We assume that Eve performs a coherent attack using an auxiliary system |E〉 and further consider it as a collective attack, applying the quantum de Finetti theorem [69]. Therefore, the state that Alice, Bob, and Eve jointly form is

(A9) $${|\phi \rangle }_{ABE}=\sum_{m,n}\sqrt{\lambda_{mn}}|\Phi_{mn}\rangle |E_{mn}\rangle ,$$

where $|E_{mn}\rangle$ is the orthogonal basis of the auxiliary system |E〉. From Equation (A8), it is clear that

(A10) $$\begin{array}{c} \lambda_{0n}=\frac{1}{Q}{\left(\sum_{l}C_{2Ml+n}\right)}^{2}, \end{array}$$

(A11) $$\begin{array}{c} \lambda_{mn}=0\;\left(m\neq 0\right). \end{array}$$

The joint system of Alice and Eve after tracing out Bob’s system can be expressed as

(A12) $$\begin{array}{cc} \rho_{AE} & ={\mathrm{Tr}}_{B}\left({|\phi \rangle }_{ABE}\langle \phi |\right)\\ & =\sum_{i}{\langle i_{B}|\phi \rangle }_{ABE}\langle \phi |i_{B}\rangle \\ & =\sum_{i,n,n^{\prime }}\sqrt{\lambda_{0n}}\langle i_{B}|\Phi_{0n}\rangle |E_{0n}\rangle \langle E_{0n^{\prime }}|\langle \Phi_{0n^{\prime }}|i_{B}\rangle \sqrt{\lambda_{0n^{\prime }}}\\ & =\sum_{i,n,n^{\prime }}\sqrt{\lambda_{0n}}\langle i_{B}|\frac{1}{\sqrt{2M}}\sum_{b_{A}=1}^{2M-1}e^{\pi i\frac{b_{A}\;\cdot \;n}{M}}{|b_{A}\rangle }_{A}{|b_{A}\rangle }_{B}|E_{0n}\rangle \\ & \;\langle E_{0n^{\prime }}|\frac{1}{\sqrt{2M}}\sum_{b_{A}^{\prime }=1}^{2M-1}e^{-\pi i\frac{b_{A}^{\prime }\;\cdot \;n^{\prime }}{M}}{\langle b_{A}^{\prime }|}_{A}{\langle b_{A}^{\prime }|}_{B}|i_{B}\rangle \sqrt{\lambda_{0n^{\prime }}}\\ & =\sum_{b_{A},n,n^{\prime }}\frac{\sqrt{\lambda_{0n}\lambda_{0n^{\prime }}}}{2M}e^{\pi i\frac{b_{A}\left(n-n^{\prime }\right)}{M}}{|b_{A}\rangle }_{A}\langle b_{A}|⊗|E_{0n}\rangle \langle E_{0n^{\prime }}|. \end{array}$$

In **Step 3’**, Alice measures the local state *A* and obtains the value $b_{A}$. Then, the state of Eve becomes

(A13) $$\begin{array}{cc} \rho_{E}^{b_{A}} & ={\mathrm{Tr}}_{A}\left({|b_{A}\rangle }_{A}\langle b_{A}|\rho_{AE}{|b_{A}\rangle }_{A}\langle b_{A}|\right)\\ & =\sum_{n,n^{\prime }}^{2M-1}\sqrt{\lambda_{0n}\lambda_{0n^{\prime }}}e^{\pi i\frac{b_{A}\left(n-n^{\prime }\right)}{M}}|E_{0n}\rangle \langle E_{0n^{\prime }}|. \end{array}$$

According to the Holevo bound, the maximum mutual information between Alice and Eve can be written as follows [70]:

(A14) $$\begin{array}{cc} \max I(A:E) & =S\left(\sum_{b_{A}}p\left(b_{A}\right)\rho_{E}^{b_{A}}\right)-\sum_{b_{A}}p\left(b_{A}\right)S\left(\rho_{E}^{b_{A}}\right)\\ & =S\left(\frac{1}{2M}\sum_{b_{A},n,n^{\prime }}\sqrt{\lambda_{0n}\lambda_{0n^{\prime }}}e^{\pi i\frac{b_{A}\left(n-n^{\prime }\right)}{M}}|E_{0n}\rangle \langle E_{0n^{\prime }}|\right)\\ & =S\left(\sum_{n=0}^{2M-1}\lambda_{0n}|E_{0n}\rangle \langle E_{0n}|\right)\\ & =-\sum_{n=0}^{2M-1}\lambda_{0n}\log\left(\lambda_{0n}\right)\\ & \equiv H_{2M}\left(E_{\mu }^{Z,D_{0}}\right). \end{array}$$

Here, S(·) is the von Neumann entropy, and $p(b_{A})$ is the distribution of $\rho_{E}^{b_{A}}$. In the assumption of an infinite block length, Alice sends bits $b_{A}\in \left\{0,1,\ldots ,2M-1\right\}$ with equal probability $p\left(b_{A}\right)=1/\left(2M\right)$.

In **Step 5**, Alice and Bob can use the multi-intensity mode to estimate the yield $Y_{2Ml+n}^{D_{0}}$ of the quantum state |2Ml+n〉 and then deduce the amount of information leakage, which is given by

(A15) $$\lambda_{0n}=\frac{1}{Q_{\mu }^{D_{0}}}{\left(\sum_{l=0}^{\infty }C_{2Ml+n}\sqrt{Y_{2Ml+n}^{D_{0}}}\right)}^{2},$$

and

(A16) $$I\left(A:E\right)=-Q_{\mu }^{D_{0}}\sum_{n=0}^{2M-1}\lambda_{0n}\log\left(\lambda_{0n}\right).$$

Finally, Alice and Bob use the QBER $E_{\mu }^{Z,D_{0}}$, the gain $Q_{\mu }^{D_{0}}$, and the inefficiency function for FEC *f* to estimate the achievable secrecy channel capacity in the case where only detector $D_{0}$ clicks:

(A17) $$R^{D_{0}}=\frac{q}{M}\;\cdot \;Q_{\mu }^{D_{0}}\;\cdot \;\left[1-fH_{2}\left(E_{\mu }^{Z,D_{0}}\right)-H_{2M}\left(E_{\mu }^{X,D_{0}}\right)\right],$$

where the coefficient q/M is the result of the mode matching in **Step 4**. The result is similar for the achievable secrecy channel capacity $R^{D_{1}}$ when only detector $D_{1}$ responds, which is

(A18) $$R^{D_{1}}=\frac{q}{M}\;\cdot \;Q_{\mu }^{D_{1}}\;\cdot \;\left[1-fH_{2}\left(E_{\mu }^{Z,D_{1}}\right)-H_{2M}\left(E_{\mu }^{X,D_{1}}\right)\right].$$

Therefore, the total achievable secrecy channel capacity *R* of protocol II, as well as that of protocol I, is given by

(A19) $$R=\max\left\{R^{D_{0}},0\right\}+\max\left\{R^{D_{1}},0\right\}.$$
